# Comparison of Serum Amyloid A Measurements in Equine Synovial Fluid With Routine Diagnostic Methods to Detect Synovial Infection in a Clinical Environment

**DOI:** 10.3389/fvets.2019.00325

**Published:** 2019-10-01

**Authors:** John David Stack, Matthieu Cousty, Emma Steele, Ian Handel, Antoine Lechartier, Tatiana Vinardell, Florent David

**Affiliations:** ^1^Institute of Veterinary Science, University of Liverpool, Neston, United Kingdom; ^2^Centre Hospitalier Vétérinaire Equin de Livet, Saint-Michel-de-Livet, France; ^3^Clinique Vétérinaire de la Côte Fleurie, Bonneville-sur-Touques, France; ^4^The Royal (Dick) School of Veterinary Studies, The Roslin Institute, The University of Edinburgh, Midlothian, United Kingdom; ^5^Clinique Vétérinaire Equine de Meheudin, Écouché, France; ^6^Equine Veterinary Medical Center, Member of Qatar Foundation, Doha, Qatar; ^7^College of Health and Life Sciences, Hamad Bin Khalifa University, Qatar Foundation, Doha, Qatar

**Keywords:** horse, septic, synovitis, serum amyloid A, specificity, sensitivity, infection, arthritis

## Abstract

Synovial fluid analysis is utilized to diagnose septic synovitis. However, not all cases are clearly and rapidly discernible with the diagnostic tools available in the laboratory. Serum amyloid A (SAA), an acute phase protein, has been shown to be elevated in synovial fluid from inflamed synovial structures. The goal of this study is to describe the correlation between two diagnostic tests measuring equine SAA levels in septic and non-septic synovial structures and to understand the correlation between an elevated SAA result and synovial sepsis. Prospective estimation of sensitivity (Se) and specificity (Sp) of two tests, handheld and ELISA, measuring SAA in synovial fluid was completed in 62 horses presented with injured synovial structures. The comparison was made to a reference diagnosis based on white cell count, percentage of neutrophils, intracellular bacteria and bacterial culture on synovial fluid. Handheld test levels were classified as: 4 lines visible—SAA level negative; 3 lines visible—SAA level mild; 2 lines visible—SAA level moderate; and 1 line visible—SAA level severe and compared to the numerical value obtained with ELISA test. The ELISA SAA test had an area under the curve of 0.88 (0.78–0.98). An ELISA cut-off of 23.95 μg/mL maximized Se and Sp. This cutoff gave a Se of 0.93 (0.66–1.00) and Sp of 0.77 (0.63–0.88). The handheld test was highly correlated with the ELISA SAA test (Spearman rank correlation 0.96) and at a cutoff of moderate or higher for positive results gave identical Se and Sp. Se and Sp of synovial fluid SAA are very reliable when clinical signs of synovitis are present for >6 h. This test, in conjunction with traditional methods, can assist practitioners to rapidly diagnose and expedite appropriate intervention of synovial sepsis.

## Introduction

Investigation of synovitis is frequently undertaken in equine veterinary practice (1). Due to the high morbidity (6–50%) and mortality (10–55%) associated with synovial sepsis (2–9), rapid differentiation between septic and non-septic synovitis should be considered a priority, so that appropriate and timely interventions can be instituted (2, 9). In cases of synovial sepsis, clinical signs can be variable depending on numerous factors such as size and species of the bacterial inoculum (4, 10), duration of infection (11), and treatment with anti-inflammatories and/or antimicrobials (12, 13). A highly sensitive and specific “gold standard” laboratory test for synovial sepsis is not currently available and will be difficult to obtain due to the complicated etiopathology of sepsis (10). Accurate classification of synovitis relies on a range of diagnostic techniques, including cytological analysis and bacterial culture of synovial fluid (4, 5, 8, 14–16). Positive bacterial culture and confirmation of intracellular bacteria within the neutrophils are two parameters considered definitive of sepsis (5, 6, 15, 17, 18). However, the Se of either parameters is low (4, 5, 17, 18), reported to be 23–31% for bacterial culture and not reported for identification of intracellular bacteria in the authors' knowledge but also low in their experience. Presumptive diagnosis of sepsis is made with cytological evidence of marked leukocytosis with neutrophilic inflammation (5–30 × 10^9^ nucleated cells/L), percentage of neutrophils from 80 to 90% and total protein above 40 g/L (4–7, 15, 17, 18). Based on the nature of the diagnostic test currently available, reporting cytological and bacteriological results can take up to 3–4 days. This delay can have severe consequences on the diagnosis, prognosis and outcome of the clinical case presented (5).

In the case of joint trauma, the acute phase response is the first inflammatory reaction acting as a barrier for pathogens and preventing further entry while decreasing tissue damage and stimulating the repair processes (19). Serum amyloid A (SAA) is an apolipoprotein with three isoforms SAA1, SAA2, and SAA3. Isoforms SAA1 and SAA2 are produced by hepatocytes and SAA3 by extrahepatic sites including synoviocytes in response to inflammatory, infectious, immunological conditions and trauma (20–22). It has a low physiological presence in the healthy horse, <5 μg/mL in normal synovial fluid, but has been shown to be elevated and rise up to 1,000-fold during acute inflammatory phases (20–22). Its increase has been reported not only in musculoskeletal diseases, septic or non-septic inflamed synovial joint conditions, but also on a variety of medical conditions including gastrointestinal and reproductive (23, 24).

Equine SAA levels, proportionate to the degree of insult, have been detected in serum (23–26) and in synovial fluid (10, 22, 25, 26). The use of a SAA handheld test has been previously reported on a model of a synovitis and septic arthritis (25) but not in a clinical environment. In the present study, clinical cases were used to evaluate the diagnostic accuracy of two tests, an enzyme-linked immunosorbent assay (ELISA) commercially available and validated in the horse and a semi-quantitative handheld test, measuring synovial fluid SAA from septic and non-septic synovial structures. We hypothesized that SAA would be significantly higher in septic vs. non-septic synovial structures and that both tests would differentiate septic from non-septic synovial structures with a high level of diagnostic accuracy. We also hypothesized that time of sampling relative to the onset of clinical signs and treatment administered prior to sampling would affect the accuracy of the SAA tests.

## Materials and Methods

### Participants

Synovial fluid samples from 62 horses, with clinical signs of synovitis (perisynovial swelling or synovial effusion) and lameness presented from August 2012 to December 2013 were included in the study. The median age of the horses was 4.5 years (range 3 days−23 years). Thirty female horses and 32 geldings were included in the study consisting of the following breeds: Irish Sport Horse (*n* = 35), Thoroughbred (*n* = 14), Standardbred (*n* = 5), pony (*n* = 3), Irish Draft Horse (*n* = 2), Cob (*n* = 2), and Quarter Horse (*n* = 1). All synovial fluid samples were harvested as part of the routine work-up of the cases. All owners consented in writing to this work-up and that samples collected could be used for research purposes.

### Diagnosis

Investigation of synovial structures consisted of physical examination, radiography, ultrasonography, synovial fluid aspiration for analysis and synovial pressure-leak testing in cases where the synovial membrane may have been breached as previously described (8, 16). Administration of antimicrobial or anti-inflammatory treatments prior to sampling was recorded. Synovial structures were given the reference diagnosis of septic (S) if they met one of the following criteria: synovial fluid positive for bacterial culture; intracellular bacteria observed on cytology; or evidence of marked neutrophilic synovial inflammation (percentage neutrophils (%N) >80%, nucleated cell count (NCC) >30 × 10^9^ nucleated cells/L) and total protein (TP) >40 g/L). Synovial structures were considered non-septic (NS) if synovial fluid was negative for bacterial culture, intracellular bacteria was not observed on cytology, and if there was evidence of mild neutrophilic synovial inflammation (percentage neutrophils (%N) <80%, nucleated cell count (NCC) <30 × 10^9^ nucleated cells/L) and total protein <40 g/L).

### Sample Processing

Synovial fluid samples were obtained by routine aseptic technique. The sample was divided into 2 EDTA blood collection vials and 1 collection in a blood culture bottle (Oxoid Signal blood culture system, Oxoid microbiological products, Thermo Fisher, Hampshire, UK) or in a plain tube if the volume available was <10 ml. Cytology was performed within 12 h on 1 EDTA sample and the following parameters determined: NCC, %N, TP, and presence of intracellular bacteria. The NCC was determined using a Neubauer chamber after treating synovial fluid with hyaluronidase solution (Sigma Aldrich, UK). The other cytological parameters were determined by examination of direct smears and cytospin samples, stained with a modified Romanowsky stain, by a board-certified clinical pathologist. TP was quantified on EDTA samples by a clinical refractometer (Atago, Japan). Bacterial culture was performed on plain samples or blood culture samples using MacConkey and blood agar. Blood culture samples were processed according to the manufacturer's guidelines. The second EDTA sample was frozen for 1–2 months at −20°C until SAA quantification.

### SAA Analysis

After thawing at room temperature, samples were subjected to 2 tests determining synovial fluid SAA levels: a commercially available multispecies ELISA validated in the horse (Accuplex Diagnostics, Kildare, Ireland) and a handheld test (EquiCheck, Accuplex Diagnostics, Kildare, Ireland). The operators processing the SAA tests were blind to the clinical signs and reference diagnosis, and blind to the results of each test. For the ELISA methodology, samples were diluted 1:500 in PBS Tween and 100 μL added along with standards and controls to a 96-well plate. All samples were analyzed in duplicate. The plate was incubated at 37°C for 1 h before washing 4 times using 300 μL of PBS Tween per well. After removal of excess wash solution, 100 μL of a ready-to-use horseradish peroxidase labeled monoclonal antibody was added to the wells and the plate incubated for a further 30 min at 37°C. The wells were then washed with PBS Tween before addition 100 μL teramethylbenzidine substrate for 10 min. The reaction was stopped by addition of 100 μL of 0.1 M sulfuric acid and the plate was read at an absorbance of 450 nm. Standards were an equine serum sample calibrated to purified equine serum SAA. Where samples were above the highest standard, these were further diluted and retested to obtain the level of SAA. The lowest detectable measure of SAA was 1 μg/mL. Results <5 μg/mL were reported as <5 μg/mL. The ELISA intra-assay variation was <2% and the inter-assay variation was <7%, indicating high performance of the test.

The handheld test, a lateral flow immunochromatographic test strip, designed for detection of SAA in whole blood samples, uses competitive assay format, without the need for sample dilution. Five microliters of synovial fluid were added to the test window, followed by addition of 3 drops of solution supplied with the test kit. After 10 min the test was interpreted by counting the visible red lines in the test window. SAA levels were determined as follows: all 4 lines visible—SAA level negative; 3 lines visible—SAA level mild; 2 lines visible—SAA level moderate; and 1 line visible—SAA level severe. If no red line was visible, the handheld test was considered invalid and was repeated.

### Data Analysis

Standards for Reporting Diagnostic accuracy studies (STARD) 2015 check list was used in this study as a guide to contribute to the completeness and transparency of reporting the diagnostic accuracy of the SAA measurement. For horses presenting more than one synovial structure harvested, just one has been chosen randomly to reduce bias in estimated confidence intervals.

ELISA SAA data was compared using Mann-Whitney U test as the values were highly right skewed. Diagnostic performance of the ELISA and handheld tests over a range of cut-off values was described using receiver operating characteristic (ROC) curves with the reference diagnosis as the “gold standard.” Area under the curve (AUC) and an estimate of its 95% bootstrap confidence interval (CI) for each ROC curve was also determined using the pROC package (10,000 samples) (27), within the R statistical system (28). A preliminary cut-off for both SAA tests was then selected, maximizing the sum of Se and Sp (Youden's Index). Using the same cut off values, test Se and Sp was calculated for synovial structures of horses that were, and were not, treated with antibiotics and/or anti-inflammatories within 48 h preceding synoviocentesis, and for horses showing clinical signs ≤6 h and >6 h. A scatterplot and Spearman's rank correlation was used to assess the correlation of both SAA tests. Uncertainty of estimates of Se and Sp were estimated with binomial exact confidence intervals. Statistical analysis using a random single limb of each horse yielded results that did not differ markedly compared to results of analysis of all structures, when treated independently.

Statistical significance was set at *p* < 0.05.

## Results

### Diagnosis

Of the 62 horses, 48 (77%) were categorized as NS and 14 (23%) as S based on synovial fluid positive for bacterial culture, intracellular bacteria observed on cytology, or evidence of marked neutrophilic synovial inflammation. Synovial fluid submitted for culture revealed 8 positives for bacteriology including *Streptococcus zooepidemicus* (x2), *Streptococcus dysagalactiae, Actinobacillus suis, Streptococcus dysgalactae, Rhodococcus equi, Escherichia coli*, Coagulase negative *Staphylococci* and 4 of those samples had intracellular bacteria detected on cytology too. Four cases were categorized as septic based on synovial inflammation only. Affected anatomical sites consisted of 43 joints, 16 tendon sheaths and 3 bursae. Twenty-eight (45%) cases had been treated with anti-inflammatories within 48 h preceding synoviocentesis (NS: *n* = 22; S: *n* = 6). Thirty-one (50%) cases had been treated with antimicrobials within 48 h preceding synoviocentesis (NS: *n* = 22; S: *n* = 9).

### SAA Levels

**Figure 1:**
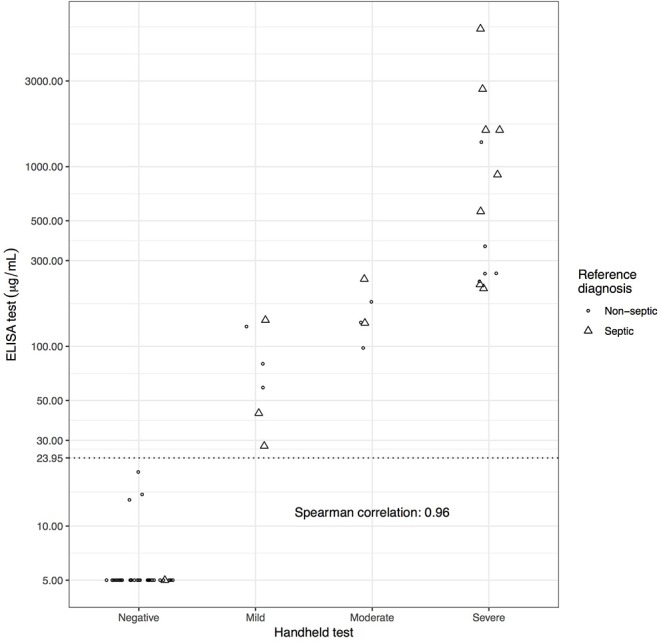
Scatterplot of ELISA SAA values vs. handheld test results with point shape representing reference diagnosis (Septic (S) synovial structures = triangles and non-septic (NS) synovial structures = circles). Points are spread horizontally where ELISA results are similar). ELISA cutoff (23.95) maximizing the sum of Se and Sp is shown as an additional labeled gridline. The Spearman rank correlation between ELISA score and ordinal handheld test score is also shown.

ELISA SAA results for the S group (mean: 1,017, median: 229, interquartile range (IQR) 1,290 μg/mL) were significantly different from the NS group results (mean: 70.2, median: 5, IQR: 11.2 μg/mL) (*p* < 0.001). Of the 48 NS structures, 37 (77%) had negative SAA on handheld, 3 (6%) had mild, 3 (6%) had moderate and 5 (10%) had severe SAA levels. Of the 14 S structures on handheld testing 1 (7%) had negative SAA, 3 (21%) had mild, 2 (14%) had moderate, and 8 (57%) had severe SAA levels. ELISA and handheld test results were highly correlated, Spearman's rank correlation 0.96 (Figure 1).

### Sensitivity and Specificity of ELISA and Handheld SAA for Prediction of Sepsis of Synovial Structures

**Figure 2:**
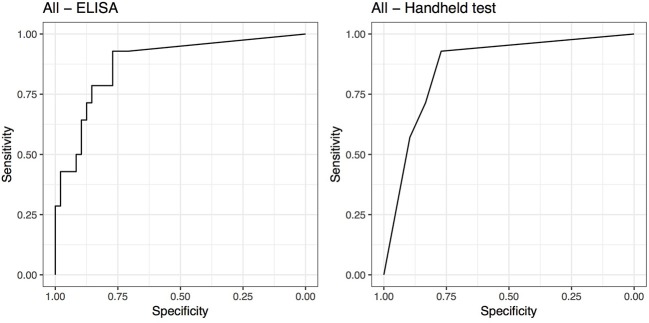
Receiver operating characteristic (ROC) curves for ELISA and handheld SAA tests for all 62 horses.

The cut-off that maximized Se and Sp for the ELISA was SAA ≥ 23.95 μg/mL [Se = 0.93 (95% CI 0.63–1) and Sp = 0.77 (95% CI 0.63–0.88)]. The AUC was 0.88 (95% CI 0.78–0.98) for the ELISA. The cut-off that maximized Se and Sp for the handheld test was SAA ≥ moderate (1 or 2 lines visible on the test strip). At this cut-off the handheld test diagnoses were concordant with the ELISA test and hence had identical Se and Sp results. The AUC was 0.86 (95% CI 0.76–0.96) for the handheld test (Figure 2).

### Influence of Synovial Sampling Timing Relative to Antibiotic and/or Anti-Inflammatory Treatment Within 48 h Preceding Synoviocentesis

**Figure 3:**
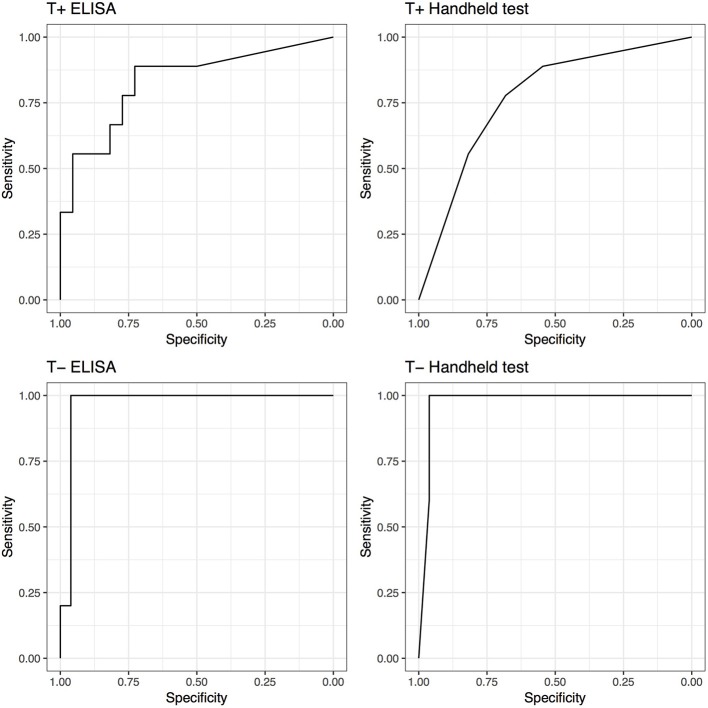
Receiver operating characteristic (ROC) curves for ELISA and handheld SAA tests for horses that received antimicrobial and/or anti-inflammatory therapy (T+) and horses that did not (T−).

Although not statistically significant, both tests demonstrated increased performance (AUC) in the untreated groups compared to treated groups. The performance of the SAA tests increased if synovial structures were not treated before being analyzed. The ELISA AUC increased from 0.83 (0.66–1) for samples treated, to 0.97 (0.91–1) for non-treated samples. Regarding the handheld test, the AUC increased from 0.77 (0.59–0.95) for samples treated, to 0.97 (0.92–1) for non-treated samples; Correspondingly, Se and Sp increased from 0.89 (0.52–1) and 0.55 (0.32–0.76) to 1 (0.48–1) and 0.96 (0.8–1), respectively. ELISA testing yielded identical improved Se and Sp (Figure 3).

### Influence of Synovial Sampling Timing Relative to Onset of Clinical Signs

**Figure 4:**
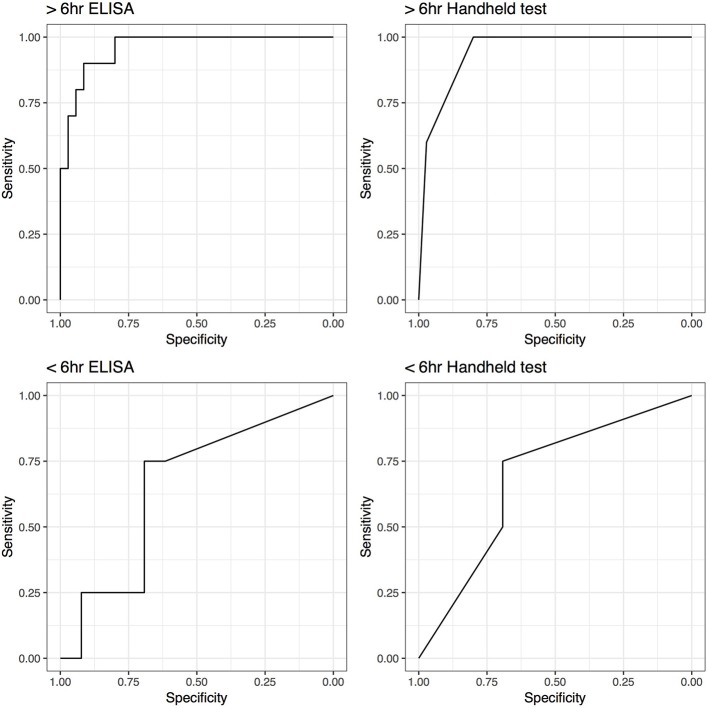
Receiver operating characteristic (ROC) curves for ELISA and handheld SAA tests for horses tested within 6 h (<6 h) and horses tested after 6 h (>6 h) of the onset of the clinical symptoms.

Unexpectedly low SAA levels (ELISA 5 μg/mL; handheld negative) were detected in 1 horse that met the septic criteria. Its joint had sustained injury within 6 h of synoviocentesis. Of the structures showing clinical signs of ≤6 h the highest SAA level was 903 μg/mL (ELISA) and severe (handheld). The performance of the SAA tests increased if synovial structures from horses sampled within 6 h of onset of clinical signs were excluded from the analysis. The AUC increased from 0.68 (0.4–0.97), for samples taken ≤6 h of onset of clinical signs, to 0.95 (0.89–1.00) for samples taken after 6 h of onset of clinical signs for the handheld test and from 0.65 (0.35–0.96) to 0.96 (0.91–1.00) for ELISA. Correspondingly, Se and Sp increased from 0.75 (0.19–0.99) and 0.69 (0.39–0.91) to 1.00 (0.69–1.00) and 0.8 (0.63–0.92), respectively for handheld test with exclusion of the structures sampled ≤6 h after the onset of clinical signs. ELISA testing yielded similar improved Se and Sp, with a minor difference in 95% CIs (Figure 4).

## Discussion

The aim of this investigation was to evaluate the diagnostic accuracy of two tests measuring synovial fluid SAA in distinguishing S from NS synovial structures. Synovial fluid levels of SAA were significantly higher in S structures compared to NS structures and both tests showed excellent Se (0.93) and good Sp (0.77) for the diagnosis of sepsis. The ELISA carries similar disadvantages as routine synovial fluid analysis, in that substantial time is required to submit, transport, analyze and report results. In contrast, the handheld test, evaluated herein, is simple to perform and provides results within minutes while the veterinarian is horse-side. The results demonstrate the potential usefulness of the handheld test as a diagnostic tool in ambulatory settings or out-of-hours in hospitals permitting early referral or appropriate treatment, while awaiting other laboratory test results.

Studies of diagnostic accuracy compare the diagnostic test, synovial fluid SAA ELISA and handheld in this case, against a gold standard diagnostic test. However, such a test, distinguishing septic from non-septic synovial structures is not currently available in horses (10). Thus, this study compares synovial fluid SAA to a reference diagnosis. To maximize the accuracy of the reference diagnosis, strict inclusion criteria were utilized for both categories. Septic structures had positive bacterial cultures, and/or intracellular bacteria, and/or marked neutrophilic synovitis and high total protein count. All S structures were treated as septic with appropriate aggressive medical and surgical interventions. Whilst 32 structures in the NS group had a high index of suspicion of sepsis due to clinical signs of effusion, heat, pain or swelling, and marked lameness in the associated limb (i.e., lame at the walk), it should be noted that for not all NS cases synovial sepsis was high on the differential list. Inclusion of these cases led to the incorporation of NS structures that had low levels of synovitis.

The AUC is a measure of the diagnostic accuracy of a test, and describes the performance of the test over all possible cut-offs. Once synovial structures sampled ≤6 h from onset of clinical signs, were excluded, the AUC of the ELISA and handheld tests were 0.96 and 0.95, respectively. Diagnostic tests with an AUC >0.9 are described as “highly accurate” (28). Analysis of the ROC curves, and consideration of the costs associated with false negative and false positive results were used to establish the cut-off values. Cut-offs were selected that maximized the sum of Se and Sp. Survival and return-to-soundness rates following synovial sepsis have been reported as 45–90% and 50–94%, respectively (2, 4–8). Thus, in synovial sepsis, ensuring a minimum number of septic synovial structures are misdiagnosed as non-septic (i.e., false negatives) is important. Higher test sensitivities would have been preferential, but due to the distribution of our data, further lowering of the cut-offs in case of the ELISA, and lowering of the cut-offs in case of the handheld test, led to a proportionately greater reduction in the Sp. Se increased to 1 when synovial structures with clinical signs of ≤6 h were excluded.

Possible explanations for septic cases revealing low SAA include: failure of the individual to mount an acute phase protein response (25); or suppression/reduction of SAA production as a result of medical therapy (22, 29). SAA production increases in response to release of pro-inflammatory cytokines IL-6, IL-1, and TNF-α (30). In humans, tetracyclines have been reported to inhibit pro-inflammatory mediators (matrix metalloproteinases, TNF-α, and IL-1) and to inhibit neutrophils and T-lymphocytes. Non-steroidal anti-inflammatories also decrease these pro-inflammatory mediators by inhibiting cyclooxygenase resulting in reduced SAA levels (19, 29, 31, 32). As an example, a chronically septic tarsal sheath had low SAA. This horse was being treated with oxytetracycline (6 mg/kg intravenously once daily) and phenylbutazone (2.2 mg/kg *per os* twice daily) when sampled. A similar mechanism of SAA “suppression” was observed for a septic middle carpal joint case. This joint was injected with corticosteroid (betamethasone 6 mg) 1 week prior to sampling.

In an experimental model of acute neutrophilic synovitis, synovial fluid SAA levels were not increased, or only mildly increased, at 4 and 8 h post intraarticular lipopolysaccharide injection, peaking at 48 h (22). It is likely that SAA in the described horse had not increased within the 6 h from injury to synoviocentesis. In light of this finding, we do not recommend the use of synovial fluid SAA testing in horses with clinical signs of ≤6 h. Synovial structures in horses with an unknown history and strong clinical suspicion of sepsis but unexpectedly low synovial fluid SAA should be retested 6 h later. Irrespective of synovial fluid SAA all open synovial structures should receive appropriate surgical and medical interventions in line with previous reports (1, 2, 4, 8, 9).

Three SAA isoforms predominate in synovial fluid: SAA 3, produced by the synovial membrane and SAA 1 and SAA 2, synthesized by the liver accessing the synovial fluid from the systemic circulation (22, 33). As neither SAA test in our study distinguished between SAA isoforms, it is possible that elevations in synovial fluid SAA, in these structures, represent systemic elevation of SAA brought about by the septic focus. Alternatively, “sympathetic” inflammatory response in the synovial structures, induced by the proximity of a septic process, may have increased local SAA synthesis (34–36). When encountering modest increases in synovial fluid SAA, where a septic process is adjacent to the synovial structure, the practitioner may choose to increase the test cut-off and thus improve the Sp of the test. Simultaneous analysis of blood and synovial fluid SAA, as well as refinement of the handheld test to specifically detect SAA 3 may be beneficial in decreasing false positives in the future.

In the clinical context, interpretation of the synovial SAA results can be challenging. For instance, SAA levels in an NS tarsocrural joint of a 4-day-old foal were moderately elevated on the handheld test. The ELISA test demonstrated a mild increase in SAA (98 μg/mL) but above the cut off (23.95 μg/mL) and would therefore have led to the conclusion of a septic process based on SAA results. This foal had pleuropneumonia and a fractured rib, both of which may have increased systemic SAA. Unfortunately, SAA measurement in the blood was not performed in this study but could have been useful in such a case.

On clinical cases, it is not uncommon to have several effused synovial structures in a close vicinity. In the current study, synoviocentesis of all effused synovial structures was performed but just one randomized anatomical structure per horse was used for the statistical analysis. In order to provide further insight on the clinical cases, it was decided to highlight the challenges associated with interpretation of the SAA results; as an example, a 5-month-old Thoroughbred with clinical signs of 3 days duration, attributable to septic osteitis of a proximal sesamoid bone, had moderate handheld SAA values and SAA of 135.5 and 144.7 μg/mL (ELISA), respectively, for the digital flexor tendon sheath and the metacarpophalangeal joint, of the same limb. Those two synovial structures adjoining the infected sesamoid bone were categorized as NS but modest increases in SAA would have led to conclude they were potentially septic. Another horse sustained a penetrating wound to the hock region and developed a latero-plantar periarticular abscess associated with tarsocrural joint infection. The tarsocrural joint was referenced as S and the tarsal sheath as NS. SAA levels in the tarsocrural joint and the tarsal sheath were 730 and 177 μg/mL, respectively. Handheld SAA levels were severe and moderate, respectively. Both synovial structures based on SAA testing would have been considered septic, while only the joint was truly infected. Focal infection can lead to false positive in the adjoining synovial structures and this should be kept into account while interpreting the results. As a clinician, assuming a synovial structure is septic and to treat it as such until proven otherwise is a very safe approach, as the consequences of not treating a false negative are greater that treating a false positive. None of the diagnostic tests including SAA measurement in the synovial fluid (ELISA or handheld) is truly “diagnostic” as a gold standard. The weight of the inadequacy of a single test to reach a diagnosis is usually reduced by performing multiple diagnostic tests assessing various angles of a condition. The results of all the diagnostic tests assessing synovial sepsis should be taken together, rather than as one conclusive test, to make reasonable clinical decisions.

## Conclusion

SAA levels were significantly higher in S vs. NS synovial structures. Overall test performance improved drastically from sufficient to excellent (37) if synovial samples were taken 6 h after the onset of clinical signs and more discreetly from very good to excellent (37) when no antibiotic and/or anti-inflammatory treatment was administered prior to synoviocentesis. A strong correlation between ELISA and handheld results was identified. Quantification of synovial fluid SAA levels via the handheld test represents an innovative and practical diagnostic tool for equine practitioners in an ambulatory setting allowing prompt diagnosis of septic synovial structures, while awaiting confirmation of the diagnosis from laboratory synovial fluid analysis and bacteriology. The handheld test may also be beneficial in referral hospitals for rapid case triage.

## Data Availability Statement

The datasets generated for this study are available on request to the corresponding author.

## Ethics Statement

The animal study was reviewed and approved by Animal Care Committee of University College Dublin Veterinary Hospital. Written informed consent was obtained from the owners for the participation of their animals in this study.

## Author Contributions

JS: study design, study execution, data analysis, drafting article, and final approval. MC: aided study design, collected samples, and reviewed the manuscript. ES: collected samples, study execution, and reviewed the manuscript. IH: statistical analysis and reviewed the manuscript. AL: collected samples and reviewed the manuscript. TV: data analysis and interpretation, drafting article, and final approval. FD: study design, sourced funding, study execution, collected samples, data analysis, drafting article, and final approval.

### Conflict of Interest

The authors declare that the research was conducted in the absence of any commercial or financial relationships that could be construed as a potential conflict of interest.
